# Major capsid reinforcement by a minor protein in herpesviruses and phage

**DOI:** 10.1093/nar/gku634

**Published:** 2014-07-22

**Authors:** Udom Sae-Ueng, Ting Liu, Carlos Enrique Catalano, Jamie B. Huffman, Fred L. Homa, Alex Evilevitch

**Affiliations:** 1 Department of Physics, Carnegie Mellon University, Pittsburgh, PA 15213, USA; 2 Department of Medicinal Chemistry, University of Washington School of Pharmacy, H172 Health Sciences Building, Box 357610, Seattle, WA 98195, USA; 3 Department of Microbiology and Molecular Genetics, University of Pittsburgh School of Medicine, Pittsburgh, PA 15260, USA; 4 Department of Biochemistry and Structural Biology, Lund University, Box 124, Lund, Sweden

## Abstract

Herpes simplex type 1 virus (HSV-1) and bacteriophage λ capsids undergo considerable structural changes during self-assembly and DNA packaging. The initial steps of viral capsid self-assembly require weak, non-covalent interactions between the capsid subunits to ensure free energy minimization and error-free assembly. In the final stages of DNA packaging, however, the internal genome pressure dramatically increases, requiring significant capsid strength to withstand high internal genome pressures of tens of atmospheres. Our data reveal that the loosely formed capsid structure is reinforced post-assembly by the minor capsid protein UL25 in HSV-1 and gpD in bacteriophage λ. Using atomic force microscopy nano-indentation analysis, we show that the capsid becomes stiffer upon binding of UL25 and gpD due to increased structural stability. At the same time the force required to break the capsid increases by ∼70% for both herpes and phage. This demonstrates a universal and evolutionarily conserved function of the minor capsid protein: facilitating the retention of the pressurized viral genome in the capsid. Since all eight human herpesviruses have UL25 orthologs, this discovery offers new opportunities to interfere with herpes replication by disrupting the precise force balance between the encapsidated DNA and the capsid proteins crucial for viral replication.

## INTRODUCTION

Herpesviruses consist of a double-stranded (ds)-DNA-filled capsid, an unstructured protein layer (the tegument) and a lipid envelope. The capsid protects the viral genome and facilitates its transport into new host cells during subsequent rounds of infection and replication. Eukaryotic and bacterial viruses (bacteriophages) retain conserved features in their replication processes despite evolutionary divergence between their respective hosts. For example, herpesviruses and dsDNA bacteriophages (1), as well as reoviruses and bacteriophage ϕ6 (2), exhibit similarities in genome packaging. Analogous to dsDNA bacteriophages, herpesviruses package their micrometer-long genome into a nanometer-scale capsid. This requires tight packaging which results in repulsive electrostatic forces between neighboring DNA helices, and the stiffness of dsDNA causes bending stress that acts on the packaged genome (3). The stressed state of packaged viral DNA generates an internal pressure of tens of atmospheres (4,5). This pressure is capable of powering genome ejection from the capsid and has been characterized for several bacterial viruses (4,6–7) and an archeal virus, His1 (8). Significantly, alterations of this internal pressure influence viral infectivity (9) and replication (10). We recently demonstrated and measured a genome pressure of ∼20 atmospheres in herpes simplex virus type 1 (HSV-1) capsids, providing the first experimental evidence of pressure within a human virus (5). Evolutionary conservation of internal pressure in viruses that infect hosts from each of the three domains of life suggests it is a key mechanism for viral infection.

In both phages and herpesviruses, DNA is packaged into the procapsid, an intermediate structure comprised of loosely assembled subunits in a spherical icosahedral shell (11–13). The weak non-covalent interactions between the protein subunits in the procapsid ensure error-free assembly and free energy minimization (14,15). However, in the final stages of DNA packaging, the internal genome pressure dramatically increases (5,16–17), which raises the central question as to how the pressurized genome is retained within the initially loosely assembled procapsid structure. In this work, we provide the first experimental evidence of a *minor* capsid protein cementing the capsid structure during the DNA packaging process. The resulting strength of the mature capsid is sufficient to withstand the internal DNA pressure and retain the genome in the capsid upon completion of packaging (18).

Herpesvirus capsids assemble from many copies of a few protein subunits in a multistage process. A number of essential minor proteins are required for DNA packaging, most of which have not yet been analyzed in detail despite their importance for viral replication (19,20). Specifically, it has been shown that minor protein UL25 (gene names will be used to refer to their respective proteins) maintains stable incorporation of the 152 kbp dsDNA genome in capsids following cleavage and packaging from a concatameric precursor (21–24). UL25 is also required for attachment of tegument proteins to the capsid (25,26) and for viral genome uncoating during the early stages of infection (27,28). It has been proposed that one of UL25's functions is similar to that of dsDNA phages’ auxiliary proteins, and may serve to mechanically reinforce capsids to prevent loss of DNA (23,29). In several dsDNA phages (e.g. λ, T4, L, ϕ29), an auxiliary protein binds to the capsid exterior during genome packaging and is required for the encapsidation of the full-length wild-type genome (30–36). The lack of this auxiliary protein leads to packaging of only partial-length genome, and in some cases capsids rupture during the course of DNA packaging (16,35). Despite years of speculation, the actual mechanical reinforcement of phage or herpes capsid by an auxiliary protein has not been demonstrated.

With this motivation, we investigate how the presence of UL25 influences the mechanical strength and stability of HSV-1 capsids using atomic force microscopy (AFM) nano-indentation technique (37–39). In parallel with these measurements, we also determine the mechanical properties of phage λ procapsids and expanded capsids with and without the auxiliary protein gpD. We provide a direct comparison between the capsid reinforcement effects of UL25 in HSV-1 and gpD in phage λ. Given the evolutionary similarities between the assembly and packaging processes in herpesviruses and phages (21,22), this comparison extends our understanding of the general principles of viral capsid assembly with post-assembly stabilization by the minor proteins.

Our results reveal that both UL25 and gpD provide a dramatic increase in the mechanical strength of the viral capsid. These findings suggest that UL25 plays a similar role in HSV-1 capsid structure as gpD does in λ: reinforcing the capsid, which facilitates packaging of the full-length herpes genome.

## MATERIALS AND METHODS

### HSV-1 capsid isolation

African green monkey kidney cells (Vero) grown in Dulbecco's modified Eagle's medium (Cellgro) with 5% fetal calf serum (GeneMate) and 5% penicillin/streptomycin (Cellgro) were infected with HSV-1 KOS strain at a multiplicity of infection of 5 PFU/cell for 20 h at 37 °C. Cells were scraped into solution and centrifuged at 3500 revolutions per minute (rpm) for 10 min in a JLA-16.250 rotor. The cell pellet was resuspended in 20 mM Tris buffer (pH 7.5) on ice for 20 min and lysed by addition of 1.25% (v/v) Triton X – 100 (Alfa Aesar) for 30 min on ice. Samples were centrifuged at 2000 rpm for 10 min and the nuclei pellet was resuspended in TNE (10 mM Tris, 0.5 M NaCl, 1 mM ethylenediaminetetraacetic acid (EDTA)) buffer with protease inhibitor cocktail (Complete; Roche). Nuclei were disrupted by sonication for 30 s. Large debris was cleared by brief centrifugation and the supernatant was spun in a 20–50% (w/w) TNE sucrose gradient at 24 000 rpm in a SW41 rotor for 1 h. The C-capsid band was isolated by side puncture, diluted in TNE buffer and centrifuged at 24 000 rpm for an additional 1 h. Capsids were resuspended in TNE and stored at 4°C. For UL25-null and UL17-null capsids, the Vero cells were infected with ΔUL25 (vFH439) (40) and ΔUL17 (41) strains, respectively. ΔUL25 strain had the full deletion of UL25 gene and yielded A and B capsids. ΔUL17 strain had the full deletion of UL17 gene and yielded only B capsids. The purification steps were the same as described for KOS strain.

### Bacteriophage λ

Bacteriophage λ procapsids and gpD protein were purified according to the protocols in ref (42,43), which were developed in Carlos Catalano laboratory. Procapsids were treated with 2.5 M urea and incubated on ice for 60 min in order to induce capsid expansion *in vitro*. The expanded capsids without gpD remain stable for some time at room temperature, at which AFM measurement was done. Next, expanded capsids were incubated with 50 μM gpD protein at room temperature for 45 min to cement the expanded capsids with gpD.

### Atomic force microscopy

All AFM measurements were performed on a MultiMode 8 AFM with NanoScope V controller, NanoScope software and NanoScope Analysis software (Bruker AXS Corporation, Santa Barbara, CA, USA). Images were acquired in Peak Force Tapping mode. All data (images and force-distance curves) were collected in liquid. A droplet of 40 μl sample was deposited on a glass cover slip. The details of substrate and sample preparations can be found elsewhere (38,44). After 30 min the sample was ready for the experiments. Rectangular gold-coated cantilevers (Olympus, Tokyo, Japan) were used. The cantilever tip radius used for measurements was 25 ± 12 nm for phage λ and 20 ± 5 nm for HSV-1. Measured averaged stiffness of cantilevers was 0.03 N/m for phage λ and 0.06 N/m for HSV-1, determined by the thermal fluctuations method (45). Spring constant and breaking force for a viral particle were obtained from the indentation measurement. At least 15–20 particles were measured to obtain each *k* and *F_break_* value. The details of spring constant calculation are described elsewhere (44). The breaking force was the maximum force a virus particle could withstand before mechanical failure, as indicated by a significant drop in the force–distance curve. The breaking force was measured directly after AFM cantilever calibration using glass surface indentation.

## RESULTS AND DISCUSSION

**Figure 1.**
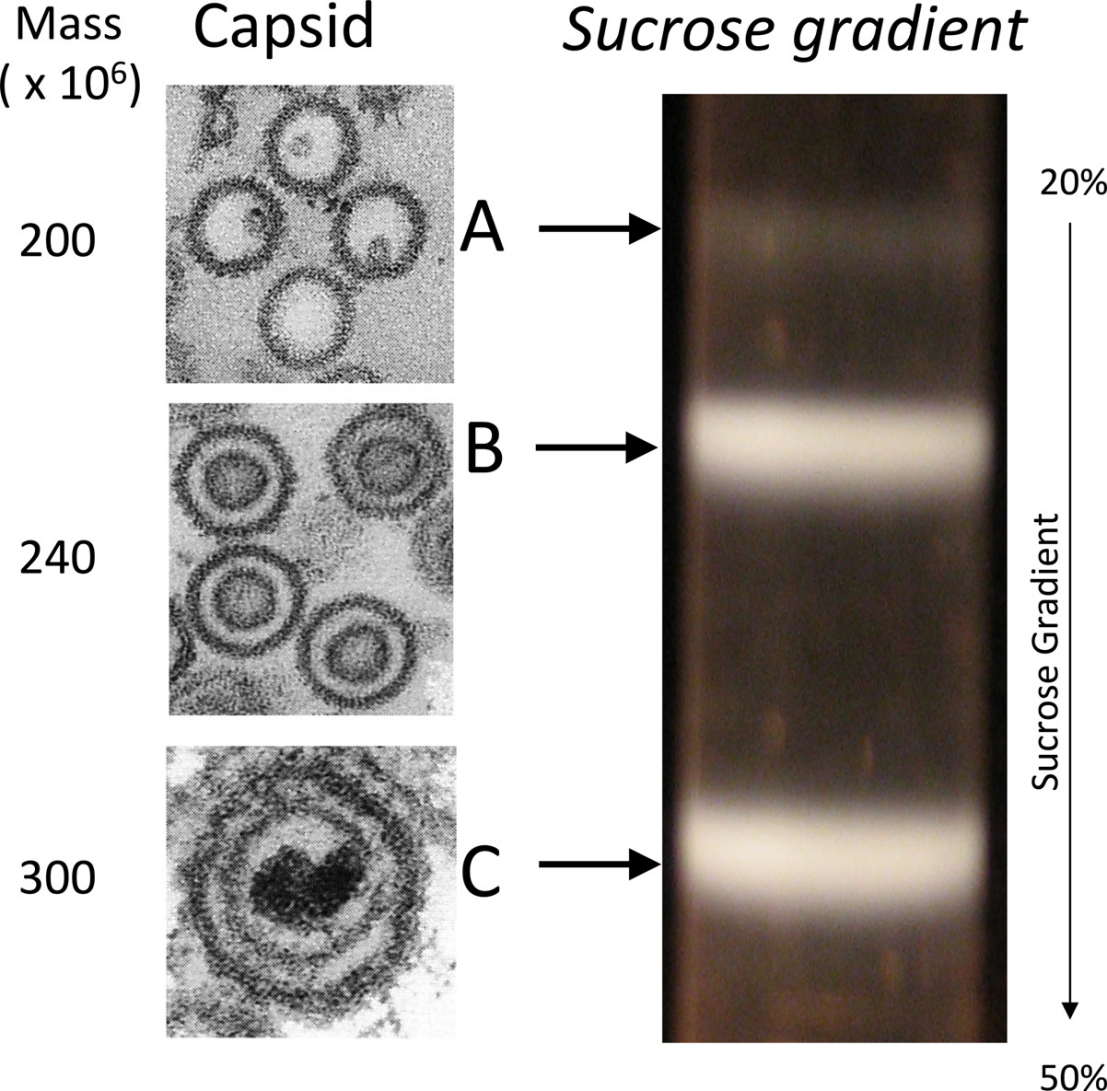
Three types of capsids found in HSV-1 infected cells. Capsid forms can be distinguished by their differential migration through a sucrose gradient or by morphology under EM (Thins sections of A and B capsids in the nucleus and C capsid within a virion).

HSV-1 is a prototypical model system to study the general infection mechanisms of herpesviruses and other eukaryotic viruses that release their genome into the cell nucleus without capsid disassembly. HSV-1 capsid assembly begins in the infected cell nucleus with procapsids assembling around a protein scaffold. The scaffold protein is proteolytically cleaved and removed during DNA packaging and DNA containing C-capsids are formed (46–48). C-capsids mature into infectious virus particles (1). Along with C-capsids, A- and B-capsids are also produced during viral replication. A-capsids lack scaffold and are thought to arise from aborted packaging and loss of the packaged DNA (1). B-capsids, which retain cleaved scaffolding proteins, do not complete the maturation pathway and are a dead-end product, see Figure 1 (1).

**Figure 2.**
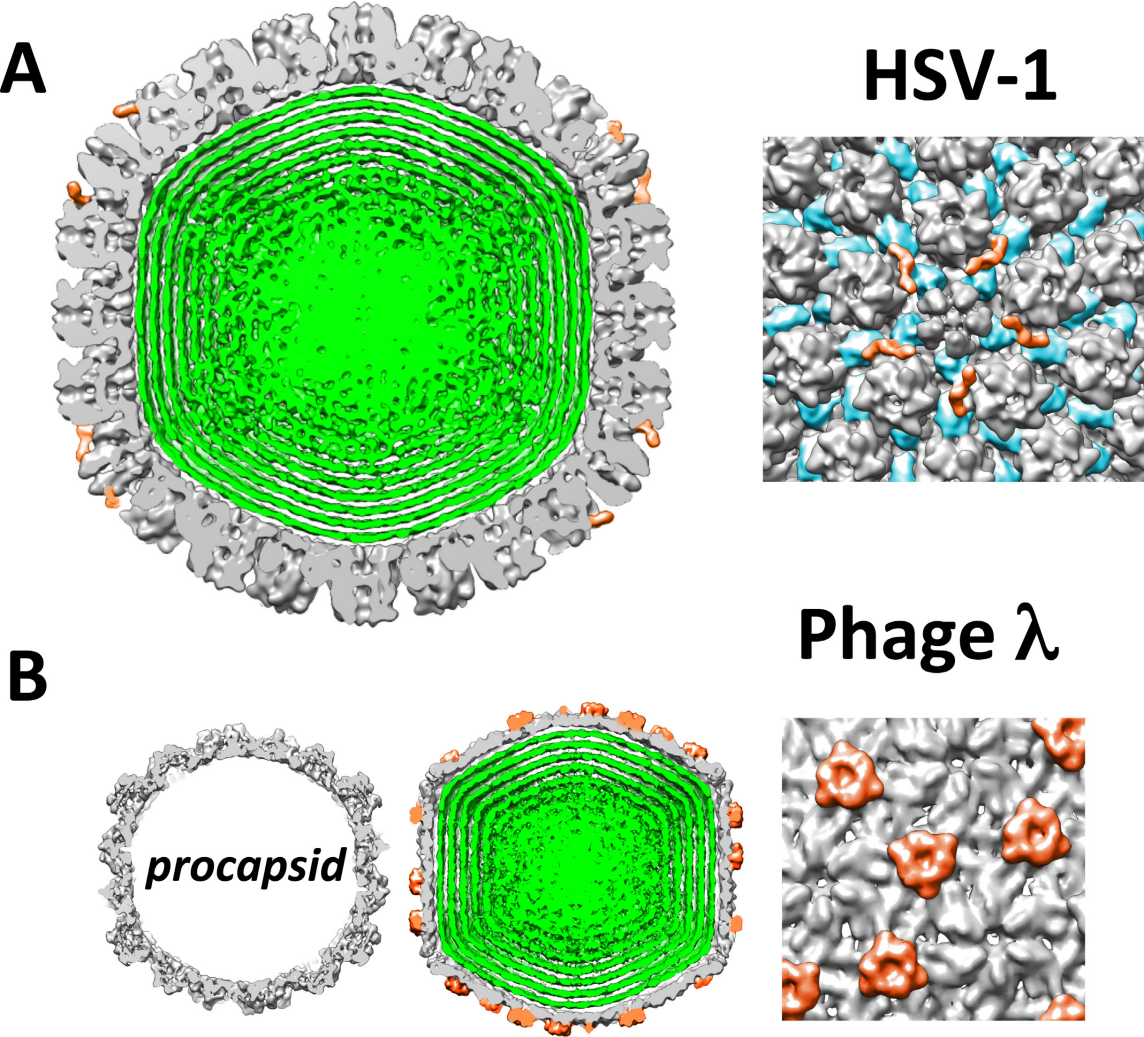
Cutaway views from cryo-EM reconstructions of (A) HSV-1 C-capsid and (B) phage λ procapsid and mature DNA-filled λ-capsid. HSV-1 and λ capsids are shown to scale. Owing to the icosahedral symmetry imposed during the reconstruction, concentrically packed DNA within the capsid becomes shells of density (green). In (A) we show five copies of UL25 (orange) surrounding each penton. The 2VP23/1VP19C triplexes are shown in blue. In (B) we show gpD triplexes (orange) stabilizing all 3-fold and quasi 3-fold axes. The density maps are downloaded from the Macromolecular Structure Database of the European Bioinformatics Institute with accession codes EMD-1354 (HSV-1) (51) and EMD-5012 (phage λ) (34).

The HSV-1 capsid is comprised mainly of the major capsid protein VP5, organized in pentameric (penton) and hexameric (hexon) capsomer subunits (49), see Figure 2A. Pentons form 11 of the 12 vertices of the icosahedral capsid structure and one vertex is occupied by the dodecameric ring of UL6 protein, which forms the portal through which DNA is packaged into the procapsid (50). This portal vertex is suggested to be sealed following cleavage of the concatameric viral DNA in the final stages of HSV-1 genome packaging. The capsomers are stabilized by the triplex heterotrimers, composed of two VP23 plus one VP19C proteins, which assemble at all 3-fold and quasi 3-fold axes (47).

Minor capsid protein UL25 is an elongated molecule that, together with UL17, forms a heterodimer (51–53). Five copies of UL25-UL17 heterodimers are found on the outer surface of each pentameric vertex as shown in Figure 2A (51). UL25 and UL17 are referred to as the ‘capsid vertex-specific component’ (CVSC) (53), and are present in A-, B- and C-capsids. Trus *et al.* proposed that conformational changes in the capsid proteins associated with DNA packaging expose UL25 binding sites, leading to higher amounts of UL25 on the surface of C-capsids compared to A- and B-capsids (54). Newcomb *et al.* (29) found that A-, B- and C-capsids contain 56, 20 and 75 copies of UL25 per capsid, respectively, although these numbers vary in the literature (54). While UL25 and UL17 are located around the penton vertices, they have been shown to form multi-subunit contacts (51–52). UL25 is located below UL17 (relative to the capsid surface) and appears to be anchored to the two VP23/VP19C triplexes closest to the penton, bridging over the hexon subunit (Figure 2A). UL17 forms a flexible interface with the upper part of UL25 pointing toward the penton (the flexibility of UL17 makes it difficult to determine its exact position with cryo-EM) (52,53). CVSC proteins may also interact with UL6 portal proteins (51).

Deletion studies have revealed seven minor viral proteins essential for DNA packaging. All but one of these deletion mutants prevent cleavage of the concatameric DNA packaging substrate and result in accumulation of scaffold containing B-capsids (53), while no A- or C-capsids are formed. In contrast, mutants lacking UL25 (29) are capable of initiating genome packaging and cleavage of concatameric DNA, but genomes are not retained within the capsids (23). This results in accumulation of unpackaged, monomeric viral genomes and empty UL25-null A-capsids (UL25-null B-capsids are also formed). These results show that UL25 is not required for DNA cleavage or activation and sustainment of the genome packaging reaction itself. Importantly, in the absence of UL25, packaged genomes can be detected that are shorter than unit length but these are minimal since they can only be detected by Southern blot analysis (55). This strongly suggests that UL25 has a crucial role either during the final steps of DNA packaging or at its completion. Many hypotheses exist for the role (s) of UL25, but its precise function during viral replication is currently not understood (23,29,40,53,55). Most importantly, its mechanical reinforcement effect on the herpes capsid structure has not been investigated.

In *Section 1* we analyze with AFM the effect of CVSC proteins (UL25 and UL17) on the capsid strength and stability. We show that UL25 provides a major reinforcement of HSV-1 capsid, while UL17 does not affect the capsid's mechanical properties. In *Section 2* we demonstrate a similar reinforcement effect on the phage λ capsid by gpD. We chose to specifically compare UL25-induced capsid reinforcement with that of gpD, since the structural details of gpD binding in λ capsid are well understood (34), which helps to explain the role that UL25 is playing in HSV-1 capsid assembly.

### UL25 reinforcement of HSV-1 capsid

**Figure 3.**
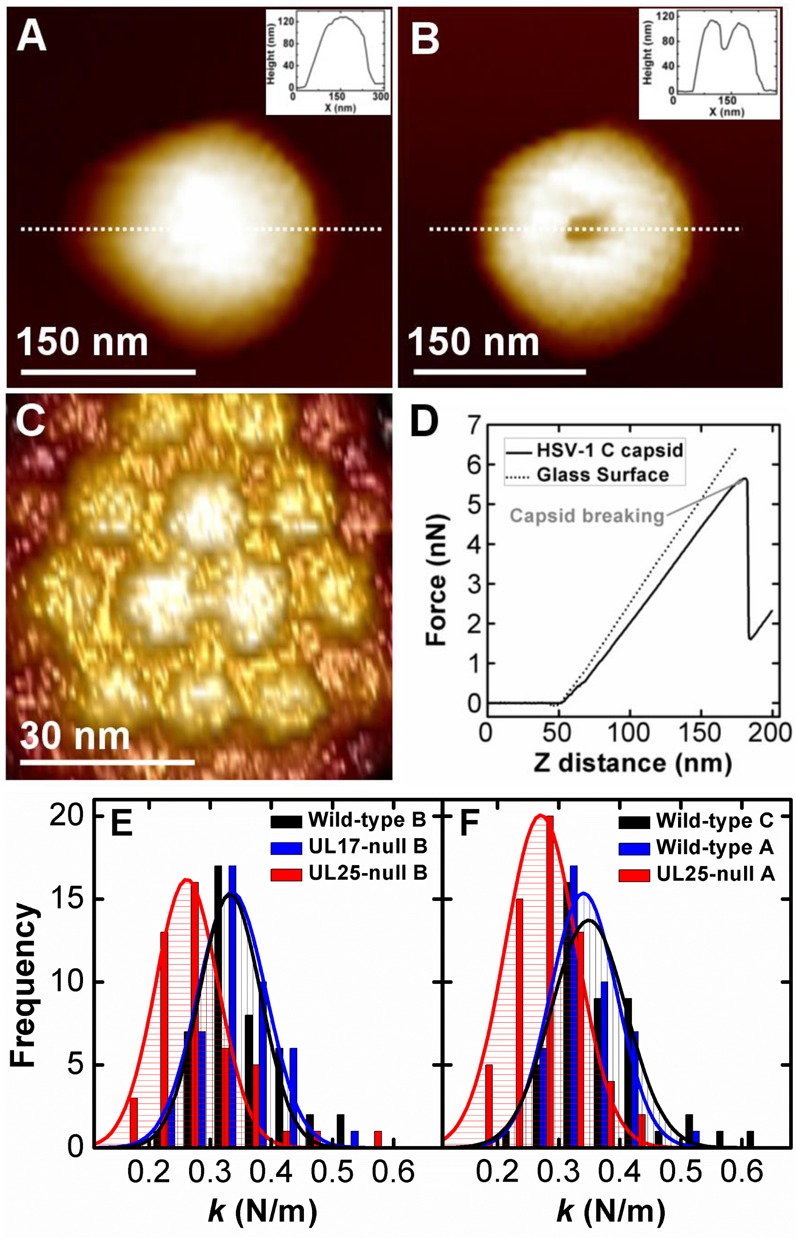
AFM imaging and nano-indentation measurements of HSV-1 capsids. (A) and (B) show AFM image of HSV-1 A-capsid before and after breaking. The insets show cross-section profiles along the dashed line. (C) Zoom-in of HSV-1 capsid with well-resolved hexons observed on the capsid surface. (D) Force–distance curves for glass substrate and for HSV-1 C-capsid. Capsid breaking is observed as a drop in the force curve. (E) Histograms of spring constants for wild-type B-capsid, UL17-null B-capsid and UL25-null B-capsid at 25°C in TNE buffer. (F) Histograms of spring constants for wild-type C-capsid, wild-type A-capsid and UL25-null A-capsid at 25°C in TNE buffer. The Gaussian fit to the data in the histograms yields the average value and the standard error shown in Table 1.

Figure 3 shows our AFM single-molecule approach for measuring the mechanical properties of viral capsids. First, individual capsids were scanned to obtain a well-resolved image, Figure 3A–C. The cantilever tip was placed at the center of the capsid surface prior to the indentation. We record the force resisting the indentation when the AFM tip is brought into contact with the capsid in solution (44). The force–distance curve in Figure 3D is linear, suggesting an elastic deformation of the HSV-1 capsid (37,38). The slope of the force–distance curve is the spring constant *k*, which describes the stiffness and stability of the viral shell. Furthermore, we also determine the maximum force at which the capsid breaks (*F_break_*) from the AFM-tip indentation, which signifies the strength of the capsid. *F_break_* is determined as the critical force value causing an abrupt drop in the linear force–distance curve (Figure 3D).

As described above, the UL25-null mutant forms A- and B-capsids (23,29) since DNA packaging is initiated but does not come to completion. The UL17-null mutant forms only B-capsids since DNA packaging fails to initiate (41,53). The angularized shells of A-, B- and C-capsids are essentially identical in structure with varying UL25 and UL17 protein content (51–52). This allows us to directly compare the measured spring constants and breaking forces for all six types of HSV-1 capsids, i.e. wild-type (wt) A-, B- and C-capsids; UL25-null A- and B-capsids; and UL17-null B-capsid. The data are shown in Table 1. All measurements were performed in TNE-buffer (10 mM Tris, 0.5 M NaCl, 1 mM EDTA) at 25ºC.

**Table 1.** Mechanical properties of HSV-1 capsids

Samples	*F_break_* (nN)	*k* (N/m)	Indentation (nm)^a^	Percentage indentation (%)^b^
WT C-capsid	5.7 ± 0.2	0.35 ± 0.01	16	13
WT A-capsid	5.3 ± 0.2	0.34 ± 0.01	16	12
WT B-capsid	3.9 ± 0.2	0.33 ± 0.01	12	9
UL25-null A-capsid	3.4 ± 0.2	0.27 ± 0.01	13	10
UL25-null B-capsid	3.0 ± 0.2	0.26 ± 0.01	12	9
UL17-null B-capsid	3.8 ± 0.2	0.34 ± 0.01	11	9
†Pentonless C-capsid	3.6 ± 0.3	0.21 ± 0.01		
†Pentonless B-capsid	3.1 ± 0.2	0.20 ± 0.01		

AFM measured breaking force (*F_break_*), spring constant (*k*) and maximum indentation distance of the capsid height in nm prior to its breaking.

^a^Indentation is calculated from the breaking force divided by *k*.

^b^Relative to the capsid outer diameter.

†Pentons are removed from capsids by 2.0 M GuHCl treatment. Data from ref. (39).

While B-capsid has lower CVSC content than A- and C-capsid, both UL17-null and UL25-null mutants form B-capsid. This allows us to compare the independent effects of both CVSC proteins on the capsid's mechanical properties. Figure 3E shows histograms of spring constants for wt, UL25-null and UL17-null B-capsid. Despite already low UL25 content, our data show that UL25-null B-capsid stiffness is decreased by ∼21% compared to wt B-capsid, and *k* drops from 0.33 to 0.26 N/m. The breaking force is reduced by ∼23% from *F_break_* ≈ 3.9 nN to 3 nN. This demonstrates that UL25 has a significant effect on both capsid stiffness and strength. The spring constant and breaking force do not need to be coupled parameters (39); in this case, however, the data suggest that the reduced strength of the UL25-null capsid (measured as *F_break_*) is also reflected in the reduction of the capsid's stiffness (measured as *k*). The decrease in the spring constant signifies that less force is required to deform the capsid to the same indentation distance. This indicates weaker capsomer–capsomer interactions when UL25 is absent.

**Figure 4.**
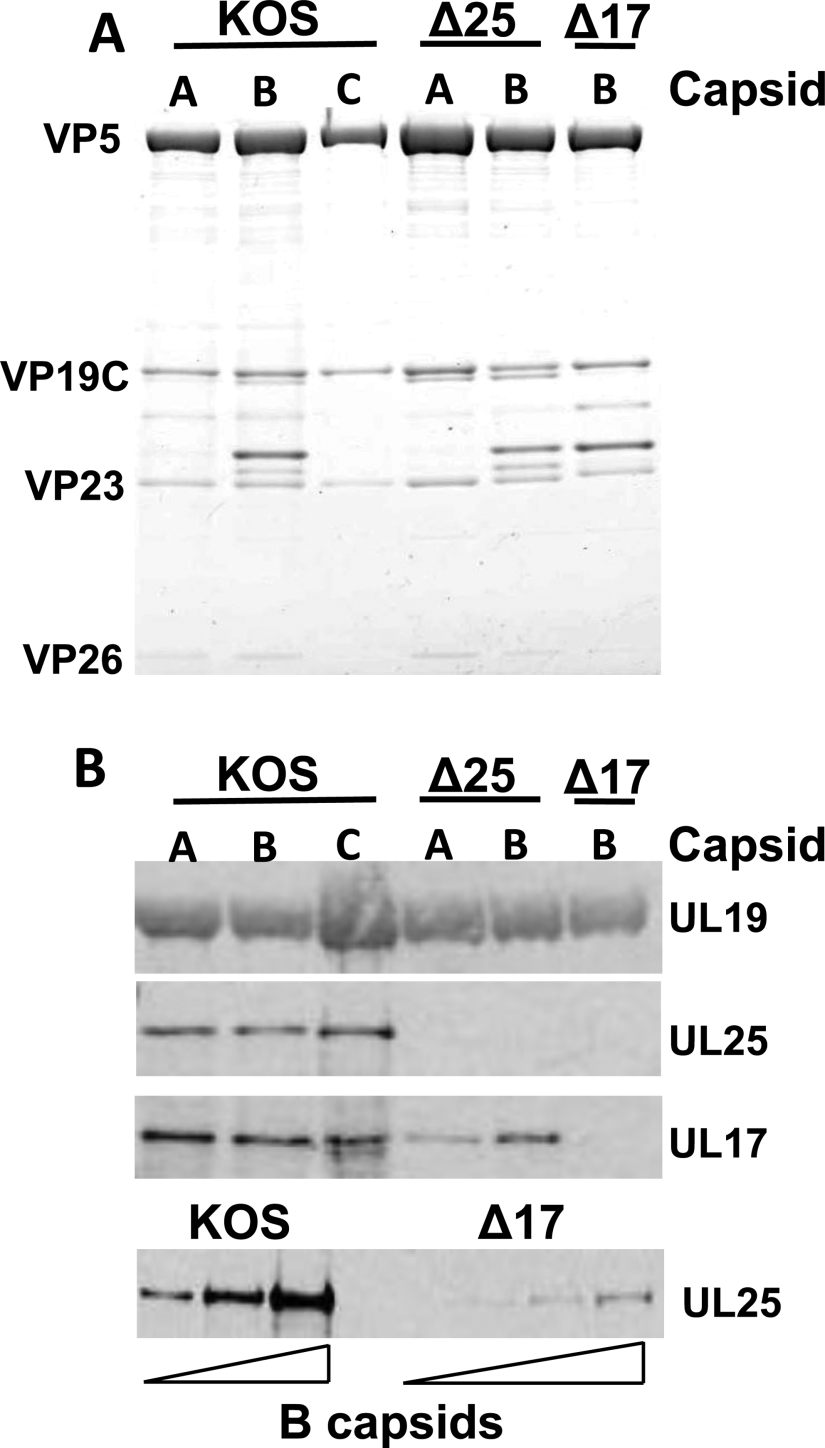
Identification of the CVSC subunits. (A) Coomassie-stained gels of KOS (wt HSV-1), UL25-null and UL17-null capsids demonstrate approximately equal amounts of sample according to capsid protein bands. (B) Western blots showing UL25 and UL17 on KOS, UL25-null and UL17-null capsids which demonstrates that UL17 is required for UL25 to bind. Conversely, UL17 binds capsids in the absence of UL25 (∼80% of the amount found with KOS capsids). (C) UL25 western blot with increasing amounts of B-capsids demonstrating that ∼5% of the amount (based on comparing scan of each band to the major capsid protein VP5) of UL25 is found with the UL17-null capsids compared to wild-type capsids.

Although UL25 and UL17 appear to form an interface with each other (52), each of them can be present on the capsid surface without the other (11,52–53). However, it has been shown that B-capsids lacking UL25 have lower UL17 content than wt B-capsids. Conversely, UL17-null B-capsids have significantly lower UL25 content than wt B-capsids (11,29,53). This is confirmed by our western blot analysis of all capsid types investigated in this work, see Figure 4. Therefore, we decided to investigate the effect of UL17 on B-capsid stability. Remarkably, UL17-null and wt B-capsid have essentially identical spring constants and breaking forces (Figure 3E and Table 1). This demonstrates that, unlike UL25, presence of UL17 on the capsid does not contribute to its stiffness and strength. Surprisingly, while UL17-null capsid contains a significantly reduced amount of UL25 compared to the wt B-capsid (11,29), this small UL25 amount appears to be sufficient to provide the UL17-null B-capsid strength similar to that of the wt B-capsid. However, it should be pointed out that the amount of UL25 in wt B-capsids is already low compared to A- and C-capsids (29). Therefore, the variations in the UL25 content between UL17-null B-capsid and wt B-capsid does not lead to significant changes in the capsid strength as observed below for comparison between UL25-null A-capsid and wt A-capsid. Furthermore, there could be other capsid stabilizing mechanisms involved, such as formation of disulfide bonds (56), which can be in a larger number in the UL17-null B-capsid, than in the other types of HSV-1 capsids. While UL17 alone does not appear to reinforce the capsid, UL25 binds more efficiently to the capsid when UL17 is present (11,29), see Figure 4. Hence, UL17 might indirectly contribute to the capsid post-assembly reinforcement mechanism by facilitating UL25 attachment.

Next we investigate how sensitive the HSV-1 capsid's stiffness and strength are to the UL25 content. UL25 content in wt HSV-1 capsids varies as B << A < C. First, we compare wt A- and C-capsid with UL25-null A-capsid. Table 1 and Figure 3F show that wt A- and C-capsid have the same spring constants (*k* ≈ 0.34 N/m for A-capsid and ≈ 0.35 N/m for C-capsid), despite the presence of DNA in the C-capsid, which was also observed in ref. (39). We have recently shown that the encapsidated DNA in the C-capsid does not contribute to the capsid stiffness, due to the counterion screening of the DNA–DNA repulsive interaction in the high salt TNE-buffer (*manuscript under review*). At the same time, the breaking force for A-capsid is slightly decreased from *F_break_* ≈ 5.7 nN (C-capsid) to ≈ 5.3 nN (A-capsid). This observation is in agreement with the ∼25% lower UL25 content in the A-capsid compared to the C-capsid (29). The most striking mechanical transformation, however, occurs when UL25 is completely removed from the A-capsid in the UL25-null mutant. The comparison between wt and UL25-null A capsid (Figure 3F and Table 1) shows a ∼21% decrease in the capsid stiffness (from *k* ≈ 0.34 N/m to 0.27 N/m) and a ∼36% drop in the breaking force (from *F_break_* ≈ 5.3 nN to ≈ 3.4 nN). This result is analogous with our observed major destabilization of the B-capsid caused by UL25 removal.

As previously mentioned, five copies of UL25 surround the pentons on the HSV-1 capsid. Pentons may experience the largest amount of radial stress in an icosahedral capsid since they are pointing outwards from the capsid surface, forming a large angle with the adjacent hexons (15). Indeed, heating A-capsid or treating it with GuHCl removes pentons along with the adjacent triplexes (including the CVSC proteins bound to them) and VP26 (a small capsid protein bound at the surface of each of the hexon VP5 subunits but not on the penton VP5 subunits), while the rest of the capsid structure remains intact (39,49,57–58). The complete removal of the pentons along with the CVSC proteins has a severe weakening effect on C-capsid stiffness and strength, see Table 1 (39). Surprisingly, removal of UL25 alone from the A-capsid has essentially the same effect on both stiffness and strength of the capsid as removal of the pentons with the CVSC (Table 1). The same is true for pentonless B-capsid compared to UL25-null B-capsid (39). These observations suggest that it is the UL25 molecules around the pentons that provide major stabilization of the HSV-1 capsid rather than the pentons themselves. Since the scaffold protein is removed in the GuHCl treated pentonless B-capsid (58) but not in the UL25-null B-capsid, this also shows that the scaffold protein does not appear to contribute to the mechanical capsid properties. Furthermore, a comparison of breaking forces between A- and B-capsids shows that B-capsid is significantly weaker than A-capsid since it has lower UL25 content (*F_break_* ≈ 3.9 nN for B-capsid versus *F_break_* ≈ 5.3 nN for A-capsid). However, the removal of UL25 yields the same capsid strength and stiffness for both UL25-null A- and B-capsid (*F_break_* ≈ 3 nN and *k* ≈ 0.26 nN), see Table 1. This demonstrates that UL25 plays a determinant role in the HSV-1 capsid stability; without UL25, A- and B-capsids are mechanically indistinguishable.

While the post-assembly reinforcement of the capsid subunits seems to be universal for many pressurized dsDNA viruses (30–36), different viral systems have evolved different ways to cement the capsid structure. For instance, we have previously shown that while phage λ uses gpD triplex as a cementing protein, the lambdoid phage HK97 does away with this extra protein and replaces it with covalent cross-links at the same 3-fold axes locations where gpD triplex binds in λ capsid (34). Also, as mentioned above, HSV-1 capsid proteins VP5, VP23, VP19C, UL17, UL25 and UL6 have been shown to form disulfide cross-links stabilizing the capsid structure (56,59). The number of cross-links appears to be higher in the capsids in the extracellular oxidizing environment (including capsids isolated in our measurements) than in the intracellular reducing environment (56). HSV-1 capsids lacking the disulfide bonds are unstable and lose pentons and peripentonal triplexes along with CVSC proteins (56). It is therefore believed that variation in the redox state in the cells during viral replication regulates different stages in the virion assembly. However, it remains unclear to what extent and how this regulation occurs.

Unlike in phage λ, the VP23/VP19C triplex subunits in HSV-1 are initially present in the procapsid structure (47) but apparently do not provide sufficient capsid strength required for DNA packaging. Instead, the subsequent binding of UL25 stabilizes the overall capsid structure and facilitates DNA packaging. UL25 molecules reinforce the capsid at its most stressed and therefore mechanically vulnerable locations around the pentameric vertices. It has been proposed that strong subunit affinity during the initial steps of capsid assembly can lead to overnucleation and formation of irregularly shaped capsids (60,61). This suggests that a post-assembly capsid reinforcement mechanism would be more favorable to ensure an error-free assembly (62).

Since herpesviruses and phages have major similarities in their DNA packaging mechanisms (22), we aim to demonstrate the universality of the post-assembly capsid reinforcement step by an auxiliary protein. In the next section, we show, for the first time, a dramatic strengthening effect of gpD triplex molecules on phage λ capsid.

### gpD reinforcement of phage λ capsid

λ-DNA is packaged in a procapsid shell consisting of the gpE major capsid protein and a portal complex situated at a unique vertex. The scaffold protein has been cleaved and removed prior to packaging. Pressure from the encapsidated DNA triggers an expansion of the procapsid, which exposes the hydrophobic E-loops between the gpE subunits (48). This allows binding of the gpD protein, forming a triplex that binds to all 3-fold and quasi 3-fold axes on the capsid (Figure 2B). We previously determined the structural details of gpD binding in the expanded λ-capsid using cryo-EM particle reconstructions (34). We also previously found that the resulting strength of mature gpD-decorated λ capsid matches the internal DNA pressure of ∼30 atmospheres and is required to withstand it (38). Despite the structural details and verification that gpD is required for complete DNA packaging (35), the gpD-induced mechanical reinforcement of the capsid has not been demonstrated.

**Figure 5.**
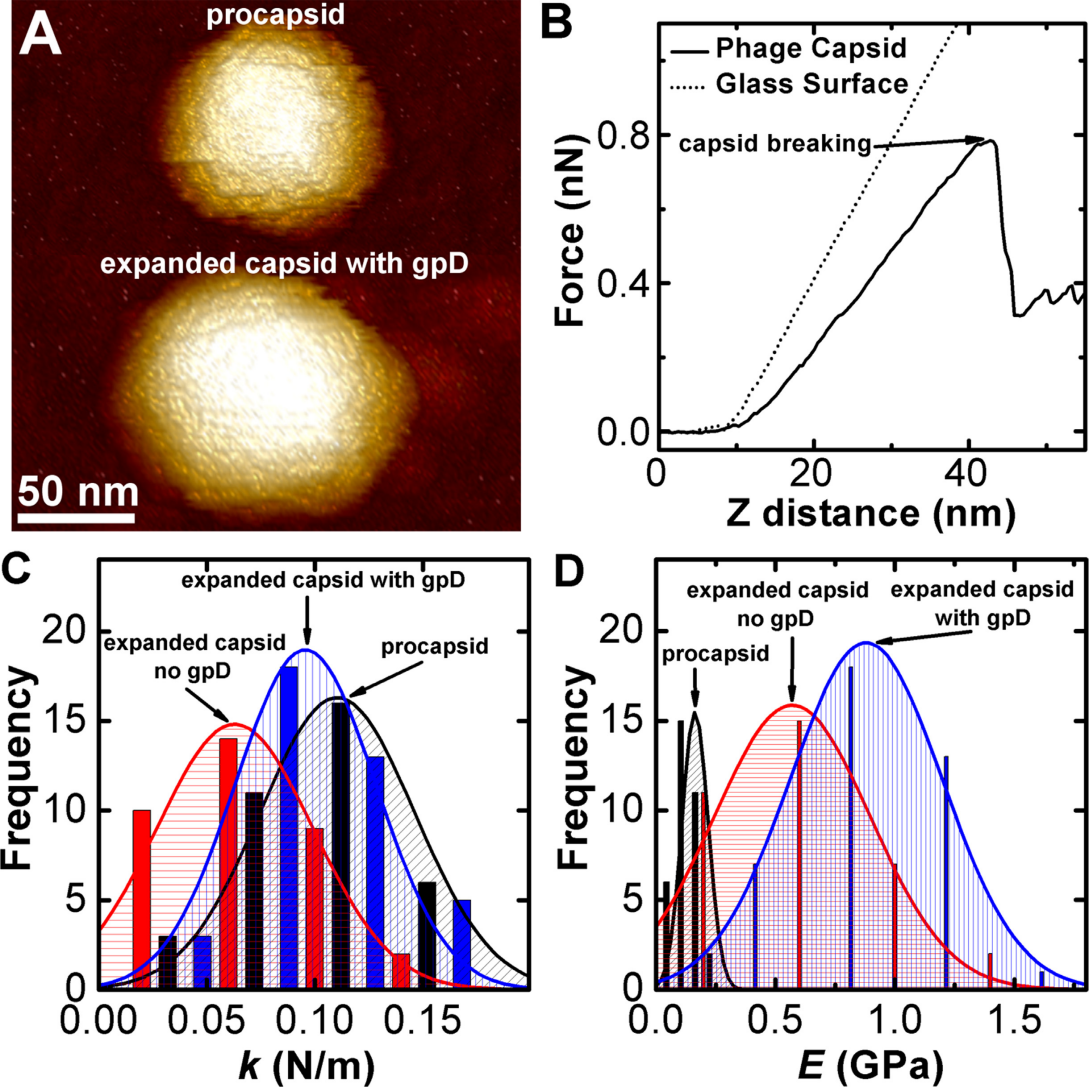
AFM imaging and nano-indentation measurements of phage λ capsids. (A) AFM image of phage λ procapsid (upper half) and 2.5 M urea expanded capsid with gpD (lower half). (B) Force–distance curves for glass substrate and for expanded capsid with gpD. Capsid breaking is observed as a drop in the force curve. (C) Histograms of spring constants for phage λ procapsid, expanded capsid without gpD and expanded capsid with gpD. (D) Histograms of Young's modulus (*E*) for phage λ procapsid, expanded capsid without gpD and expanded capsid with gpD. The Gaussian fit to the data in the histograms yields the average value and the standard error shown in Table 2.

λ-procapsid expansion can be triggered *in vitro* by 2.5 M urea treatment (43). The resulting capsid becomes angularized, its volume is almost doubled and the wall thickness is decreased from ∼4 to ∼1.8 nm (34), see Figure 2B. Purified gpD is added after the capsid expansion to form the mature λ-capsid. Using AFM nano-indentation we show the crucial role that gpD triplex molecules play in stabilizing the expanded λ capsid. Analogous to our HSV-1 analysis, we measure the spring constant (*k*) and the breaking force (*F_break_*) for λ procapsid and expanded λ-capsid with and without gpD (Figure 5). The force–distance curves for all three types of λ-capsids are linear, suggesting an elastic deformation, where the slope is *k*. All phage λ data are summarized in Table 2.

**Table 2.** Mechanical properties of phage λ capsids

Samples	*F_break_* (nN)	*k* (N/m)	*E* (GPa)	Indentation (nm)^a^	Percentage indentation (%)^b^
(1) Procapsid	0.48 ± 0.05	0.11 ± 0.01	0.16 ± 0.01	4.4	8
(2) Expanded capsid no gpD	0.45 ± 0.05	0.06 ± 0.01	0.57 ± 0.06	7.5	12
(3) Expanded capsid with gpD	0.78 ± 0.05	0.10 ± 0.01	0.88 ± 0.05	7.8	12

AFM measured breaking force (*F_break_*), spring constant (*k*), Young's modulus (*E*) and maximum indentation distance of the capsid height in nm prior to its breaking. *E* is calculated from *k* using thin shell continuum model approximation (38,63).

^a^Indentation is calculated from the breaking force divided by *k*.

^b^Relative to the capsid outer diameter.

Figure 5C shows histograms of the spring constants for all three types of λ-capsids. In a separate set of control measurements, we confirmed that the expanded capsids with gpD have the same *k* and *F_break_* values in 2.5M urea as the expanded gpD decorated capsids in Tris-Mg-buffer alone (data not shown) [2.5 M urea was added to the Tris-Mg-buffer used for procapsids]. Therefore, the observed change in the capsid mechanical properties is associated only with the expansion and not with the urea addition. Our data show that λ-capsid expansion leads to a spring constant decrease by ∼2 times from *k* ≈ 0.11 to 0.06 N/m. However, when gpD is added to the expanded capsids, the spring constant returns to its initial value of *k* ≈ 0.10 N/m. The same *k*-value was obtained for empty wt phage λ capsid (phage ghosts), where the empty shells have been emptied of their genetic content after maturation (18).

Because of significant changes in λ-capsid geometry, we could not do a relative comparison of the capsid's mechanical properties based on the stiffness change associated with the expansion step. Instead, we use the thin shell approximation (63) to compare the capsid stiffness based on the measured spring constant. Finite element simulations (64) have confirmed that this continuum mechanics model describes well the stiffness of many viral capsids (including phage λ) (38,44). The capsid stiffness is provided by the elastic modulus *E* (Young's modulus), which normalizes the spring constant value with the shell radius and the wall thickness. The spring constant is given by *k = αEh^2^/R*, with shell thickness *h*, capsid radius *R* and a proportionality factor α which to a good approximation is equal to 1 (38,44,64–65). Figure 5D shows histograms of Young's moduli *E* obtained from the spring constant data. The figure reveals that, despite the expansion and significant thinning of the capsid wall, the capsid stiffness increases by ∼2.5 times (*E* increases from 0.2 to 0.5 GPa). Apparently, the conformational changes in gpE subunits, resulting in expansion and angularization of the capsid, also increase the shell's rigidity. When gpD protein is added to the expanded capsid, Young's modulus is further increased by ∼1.8 times to *E* ≈ 0.9 GPa. Thus, during the process of DNA packaging, transition from procapsid to mature λ-capsid leads to a total increase in stiffness by ∼4.5 times, suggesting a dramatic increase in stability of the viral shell.

In a parallel experiment, with capsid expansion without gpD, the breaking force remains essentially unchanged, *F_break_*(procapsid) ≈ 0.48 nN and *F_break_*(expanded capsid without gpD) ≈ 0.45 nN, see Table 2. With gpD addition, however, the breaking force is increased by more than 70% to *F_break_*(expanded capsid with gpD) ≈ 0.78 nN. These observations demonstrate a major reinforcement of the λ-capsid resulting from capsid expansion and gpD binding in response to DNA packaging.

## CONCLUSIONS

One of the general principles of viral capsid assembly is the formation of weak bonds between the capsid subunits. This provides an error-free assembly and minimizes the free energy (15,66). Paradoxically, dsDNA motor-packaged viruses require the capsid structure to be sufficiently strong to withstand internal genome pressures of tens of atmospheres (4,5). Our results reveal how viruses resolve these two contradicting requirements by first assembling a looser procapsid structure, which later, in the course of DNA packaging, is reinforced by the addition of a minor capsid protein that stabilizes the capsomer interactions.

We show that binding of UL25 in HSV-1 provides similar capsid reinforcement as gpD binding in phage λ: the breaking force for both herpes and phage capsids is increased by ∼70% [obtained from *F_break_* (C-capsid) / *F_break_* (UL25-null A-capsid) and *F_break_* (expanded λ-capsid no gpD) / *F_break_* (expanded λ-capsid with gpD)]. We also demonstrate that it is only the UL25 protein that reinforces the HSV-1 capsid, not the whole CVSC complex around the pentons; the UL17 portion of CVSC does not contribute to the reinforcement effect. However, since UL17 is required for efficient binding of UL25 to the capsid, it indirectly controls the extent of capsid stabilization and can potentially regulate the gradual capsid reinforcement process associated with progression of DNA packaging. This hypothesis is supported by observations that UL17 is required at the early stage in DNA packaging (41), while UL25 functions later in the packaging process (11,23,55). Timed capsid stabilization occurs in phage λ. gpD binding sites are gradually exposed at the triplexes as a result of λ procapsid-to-capsid expansion from the pressure built-up during DNA packaging (34–35,56). While HSV-1 procapsids do not undergo expansion, UL17 facilitated UL25 binding and disulfide bond formation could contribute to timed stabilization (56). UL25 and gpD have an analogous effect on the increase in capsid strength, which suggests that post-reinforcement of the loosely assembled capsid is an evolutionarily conserved mechanism required for successful genome packaging.

The HSV-1 capsid is significantly larger than the λ capsid (*T* = 16 versus *T* = 7) and withstands lower internal pressure than the λ-capsid (18 atm in HSV-1 versus 25 atm in λ) (5,67). It is interesting to note that HSV-1 requires only 75 copies of UL25, while the smaller λ-capsid requires 420 copies of gpD (34) to achieve a similar capsid reinforcement effect. HSV-1 does away with hundreds of copies of UL25 by providing stabilization only at the pentons, where the capsid is mechanically most fragile (29,57). The HSV-1 triplexes composed of 2VP23/1VP19C serve to support the hexamers located at the 3-fold and quasi 3-fold axes of symmetry on the HSV-1 capsid. gpD, on the other hand, stabilizes the whole λ structure (Figure 2). This difference in capsid reinforcement tactics between HSV-1 and λ is supported by the fact that high radial stress is more concentrated around the pentons in the larger capsid structures, such as HSV-1, requiring presence of stabilizing UL25 protein at the pentons. At the same time, in the smaller capsids, such as phage λ, the stress is more homogeneously distributed over the capsid surface, requiring gpD stabilization of the entire capsid (15). Furthermore, it can be noted that smaller capsid structures (smaller *T* number) have usually more spherical shape than larger capsids (larger *T* number), which are more faceted with a pronounced icosahedral symmetry (68).

In conclusion, this work establishes that UL25 provides major reinforcement of the HSV-1 capsid, which is required for retention of the pressurized herpes genome. Since all of the eight human herpesviruses have orthologs of UL25 (69), developing ways to perturb the force balance between the internal DNA pressure and the capsid strength could present new classes of anti-viral treatments interfering with Herpes replication.
